# A review of the 8th edition of the AJCC staging system for oropharyngeal cancer according to HPV status

**DOI:** 10.1007/s00405-020-05979-9

**Published:** 2020-04-27

**Authors:** Piotr Machczyński, Ewa Majchrzak, Patryk Niewinski, Joanna Marchlewska, Wojciech Golusiński

**Affiliations:** Head and Neck Surgery Department, The Greater Poland Cancer Centre, University of Medical Sciences Poznan, 15 Garbary St, 61-866 Poznan, Poland

**Keywords:** Oropharyngeal squamous cell carcinoma, HPV-related oropharyngeal cancer, AJCC staging system, TNM staging system

## Abstract

**Background:**

The incidence of oropharyngeal squamous cell carcinoma (OPSCC) has increased substantially in recent decades, particularly p16-positive human papillomavirus (HPV)-related OPSCC, which has risen by 50% in western countries. HPV-positivity is the most favourable non-anatomic predictor of oropharyngeal cancer outcomes, which underscores the importance of incorporating this variable into the cancer staging system.

**Methods:**

In the present article, we review the differences between the 7th and 8th editions of the AJCC staging system, with particular focus on the role of HPV-positivity in patients with head and neck cancer.

**Results:**

In the previous edition (7th edition) of the AJCC/UICC manual, HPV status and its correlation with nodal metastasis were not considered, thereby leading to incorrect lymph node (N) staging and, potentially, inadequate treatment and worse outcomes. The 8th edition of the AJCC manual addresses these issues, providing more accurate discrimination between groups and better risk stratification in patients with HPV-positive OPSCC. In the future, additional adjustments are likely to be needed, such as unification of the pathological and clinical staging models.

**Conclusions:**

The new staging system is substantially more accurate than the previous system and should be widely adopted in routine clinical practice.

## Introduction

Oropharyngeal squamous cell carcinoma (OPSCC) is a common type of head and neck cancer. In 2018, nearly 93,000 patients were diagnosed with OPSCC, accounting for more than 13% of all head and neck cancers globally [1]. OPSCC has traditionally been associated with two main, synergistic risk factors, tobacco use and excessive alcohol consumption [2]. The main reason for the unremitting rise in the incidence of OPSCC in recent decades is human papillomavirus (HPV) infection [3]. Approximately 50% of OPSCC cases in western countries are HPV-positive, with higher rates in the United States and Scandinavia, and lower rates in Southern Europe [4]. Risk factors include: numerous oral and vaginal sexual partners, young age of sexual initiation, and in men a history of anogenital warts, all of which may lead to viral colonization of the oral and oropharyngeal mucosa. Immunocompromised patients, sexual partners of women with cervical cancer, and patients with a history of HPV-related anogenital cancer are at increased risk of acquiring HPV-positive head and neck cancer [5].

Non-routine pathological diagnostic tests are necessary to determine HPV-positivity. P16 immunohistochemistry (IHC), which has been in clinical use since 2003, is a widely-accepted diagnostic technique for HPV-driven head and neck cancer [6]. According to the findings of a recent study, p16 immunohistochemistry is highly sensitive but only moderately specific when used as a single test to determine HPV-positivity in oropharyngeal cancer [7]. Other tests, such as DNA- and mRNA-based polymerase chain reaction (PCR) and in situ hybridization (ISH), are also widely used, often in combination with other techniques based on recommendations to use bimodal diagnostic approaches for greater accuracy (e.g., p16 staining combined with HPV DNA or mRNA detection).

HPV-positive OPSCC is associated with lower T stage and higher N stage compared to HPV-negative OPSCC [8]. [9] Early nodal involvement can be attributed to the lack of subepithelial connective tissue layers underneath the tonsillar crypt epithelium. Thus, tumor cells from the layer of basal cells move to the neck lymph nodes before the primary lesion has grown large enough to be detected on a routine examination. For this reason, up to 40% of HPV-positive OPSCCs are diagnosed with ipsilateral neck involvement [10].

HPV positivity is the most favourable non-anatomic predictor of oropharyngeal cancer outcomes. HPV-positive OPSCC is highly responsive to anti-cancer treatments, resulting in better overall survival (OS) and higher rates of locoregional control [11]. The oncogenic mechanisms underlying HPV-positive OPSCC differ from those in tobacco and alcohol-related HPV-negative OPSCC; consequently, conventional staging systems that fail to consider HPV status do not provide an accurate estimate of the patient’s true prognosis [12]. Given the continuous rise in the prevalence of HPV-positive OPSCC, many specialists have advocated far-reaching changes—rather than simply an update—to the cancer staging system.

## History

It became clear decades ago that a comprehensive classification system for cancer was needed to facilitate treatment selection and to determine prognosis and risk stratification. The unified cancer staging system, based on an assessment of three main characteristics—primary tumor size and extension (T), nodal advancement (N) and distant metastasis (M)—was developed by special committee chaired by Pierre Denoix at the Institut Gustave-Roussy in Villejuif, France in 1944 [13]. That staging system was adopted by the International Union against Cancer (UICC), which was created and established by members of the committee in 1954. In 1977, the American Joint Committee on Cancer (AJCC) published an independent manual for cancer staging. The first unified edition of the UICC/AJCC classification was published in 1987. The unified system eliminated surgical staging (sTNM) and revised the lymph node classification (N) to avoid the subjectivity of fixation, which was replaced with an assessment of the size and number of involved lymph nodes [14]. This classification has been updated numerous times, with the 5th, 6th, and 7th editions published, respectively, in 1997, 2002, and 2009 [15].

The TNM staging was designed to be user-friendly; indeed, its simplicity is one of its main advantages, making it the most widely accepted and used cancer classification system in clinical use. Even though a more complex system could, theoretically, be more accurate, any additional complexity would likely not be well-accepted in clinical practice, leading to poor compliance. This is why less important prognostic data are not included in the TNM staging system. Each new edition of the AJCC/UICC manual includes changes designed to improve the accuracy and usability of the system, thus increasing compliance. The latest edition of the AJCC staging system—the 8th edition—for oropharyngeal cancer staging was based on the International Collaboration on Oropharyngeal Cancer Network for Staging (ICON-S) study, which incorporated data from five North American and two European centres. This includes an entirely new staging system for HPV-positive oropharyngeal cancer [16].

## The 7th edition of the AJCC staging system: a need for change

Given the markedly better prognosis of HPV-positive OPSCC, it became clear that the 7th edition of the AJCC cancer staging system was inadequate in terms of risk stratification and prognosis. HPV-positive OPSCC patients with early lymph node involvement often present with bulky neck lymph nodes, even if the primary tumor is not well-advanced. Based on the criteria set out in the 7th edition, up to 80% of OPSCC patients were diagnosed with stage IV disease [11].

Taberna et al. compared four different staging classification systems, including the 7th edition of the AJCC system and an ICON-S proposal (which was eventually accepted for inclusion in the 8th edition of the AJCC manual), to classify 66 HPV-positive OPSCC patients. Compared to the 7th edition, all the other staging systems provided a more accurate overall survival estimate and better between-group discrimination. Interestingly, the ICON-S model performed better when more than one biomarker (i.e., HPV-DNA plus p16 or HPV-DNA plus HPV-mRNA was used to define HPV-positivity [17].

In a study of 1204 patients, Nauta et al. demonstrated important limitations in the 7th edition of the manual. Contrary to expectations, those authors found that 5-year OS was lower in stage I–II disease than in stage III–IV disease [18]. Miccio et al. evaluated 3407 pathologically node-positive HPV-OPSCC patients, showing that the N variable of the 7th edition did not affect OS as expected: compared to N0 patients, the only N group with significantly worse survival was N2c, while OS in patients with stage N1, N2a, N2b or N3 was comparable to the rates observed in stage N0 patients [19]. Han et al. compared the 7th and 8th editions in a study involving 736 HPV-positive patients with OPSCC. When the 7th edition criteria were applied, they found no differences in OS among stage pN0, pN1 and pN2 patients [20]. Considering the findings described above, there was a clear, urgent need to re-evaluate and modify the staging system in the 7th edition to include HPV status, which would help to ensure adequate discriminative power between the different stages. The latest edition of the UICC/AJCC manual (8th edition), which now includes a new staging system for HPV-associated oropharyngeal cancer, was published in 2017 and came into effect in January 2018 [21].

## The 8th edition of the AJCC staging system: changes in OPSCC staging

The 8th edition of the TNM staging manual included several important changes. New chapters on head and neck skin cancers and unknown primary carcinomas were added and the classification system made major changes to OPSCC staging according to HPV status. In the 8th edition, HPV positivity is determined by p16 testing of tumor tissue; cases with at least moderate staining intensity and diffuse staining (≥ 75% of tumor cells) are now classified as probable HPV-associated etiology based on p16 positivity [22].

The main differences from the 7th edition with regard to oropharyngeal squamous cell carcinoma are as follows:

Tis (in situ) is not included in p16-positive oropharyngeal cancer; the T0 category is only used in p16-positive metastatic lymph nodes and has been reassigned to a new chapter (unknown primary carcinoma), as the primary tumor is presumed to be oropharyngeal cancer. In p16-positive oropharyngeal cancer, stages T4a and T4b are now unified in a single category (T4).

With regard to clinical N staging for p16-positive OPSCC, ipsilateral lymph nodes (one or multiple) no larger than 6 cm are characterized as N1. Bilateral or contralateral nodes are stage N2, provided none are larger than 6 cm. No subcategories are included in the N2 stage. Nodes larger than 6 cm are classified as N3.

For p16-negative OPSCC, changes to clinical N staging apply only stage N3, which is now divided into two subcategories: stage N3a if the nodes are larger than 6 cm but without extranodal extension; and stage N3b if there are any signs of extranodal extension, either clinical or radiographic.

Pathological N staging is applicable only to surgically-treated patients. While this remains the same as in the 7th edition for p16-negative OPSCC, it differs widely for p16-positive OPSCC: stage N1 is defined as involvement of no more than four metastatic lymph nodes (laterality is not considered) and stage N2 is defined as more than four metastatic nodes. Stage N3 has been removed from the pathological staging.

## Validation of the 8th edition of the AJCC staging system: oropharyngeal cancer

Several studies have compared the 7th and 8th editions of the TNM manual in the same group of OPSCC patients, with the stage modified according to HPV status. Park et al. re-evaluated 188 patients with HPV-positive OPSCC. In that study, using the 7th edition criteria, more than 85% of patients were classified as stage III and IV (19.1% and 67.6%, respectively) whereas most patients were classified as stage I and II using the criteria in the 8th edition (76.1% and 20.7%, respectively). Only eight patients (4.3%) were classified as stage I according to the 7th edition criteria. By contrast, none of the patients were classified as stage IV under the criteria of the 8th edition [23]. The same study compared both groups in terms of the recurrence rates: under the 7th edition criteria, these rates were 0%, 11.8%, 2.8%, and 11.8%, respectively, for stage I, II, III, and IV patients; in contrast, following the criteria of the 8th edition, recurrence rates were 8.4%, 10.3%, and 33.3%, respectively, for stages I, II, and III. These findings show that use of the 8th edition criteria resulted in a higher recurrence rate, which implies better between-group discrimination. Similar results were reported by Sharma et al., who also classified a group of OPSCC patients (*n* = 621) using criteria from both the 7th and 8th editions. Using the 7th edition criteria, only 7.9% of patients were stage I or II; by contrast, when the 8th edition criteria were applied, this percentage rose to 62.9% [24]. Jacobi et al. re-evaluated 137 patients with OPSCC treated with primary surgery. Under the 7th edition criteria, most patients were classified as stage III and IV (19% and 62%, respectively); however, when the criteria from the 8th edition were applied, 95% of HPV-positive patients were categorized as stage I or II [25]. Zhan et al. evaluated 3745 patients with OPSCC. Following the criteria in the 7th edition, only 10.2% of patients were classified as stage I and II (3.6% and 6.6%, respectively) while more than 95% of cases were classified as early stage (I or II) when the 8th edition criteria were applied (80.2% and 17.7%, respectively). Interestingly, the percentage of patients with pN1 disease increased from 17.3% (7th edition criteria) to 75.9% in the 8th edition [26].

As those studies show, the change in criteria resulted in patients being reassigned to different stages, resulting in improved discrimination. However, survival rates also differed widely when the two set of criteria were compared. Mizumachi et al. analyzed 195 patients with OPSCC, showing that no survival differences (7th edition) between stage I–II and stage III–IV patients with p-16 positive oropharyngeal cancer. By contrast, when the 8th edition criteria were applied, patients with stage III disease presented significantly worse survival—as would be expected—than stage I–II patients. In that study, of the 80 stage IV patients (7th edition criteria), 45 were reassigned to stage I and 16 to stage II when the criteria from the 8th edition were applied [27]. Similarly, Haughey and Sinha found that pathologic TNM staging was a prognostic factor for both disease-free survival (DFS) and disease-specific survival when the 8th edition criteria were used, but not prognostic when the 7th edition criteria were applied [28, [29].

## Controversies

P16 immunohistochemistry is recognized in the 8th AJCC TNM classification as a standard method to evaluate HPV-driven carcinogenesis in oropharyngeal cancer. Studies have shown that positive p16 immunohistochemistry may reflect only a transient infection and thus this variable may be insufficiently specific to be considered a reliable diagnostic tool on which to base treatment de-intensification [30]. Moreover, when more than one method is used to evaluate HPV-positivity, the results frequently differ. Larsen et al. found that 5% of patients with oropharyngeal cancer were HPV DNA positive but p16 negative while 9% were HPV DNA negative yet p16 positive [31]. It appears that p16−/HPV+ findings may reflect a bystander infection or contamination rather than a true HPV that impacts the carcinogenetic process. Similarly, p16+/HPV− findings may be unrelated to HPV infection, but rather associated with other genetic perturbations [32].

Rietbergen et al. reported that patients with p16+/HPV− OPSCC had worse OS than patients with p16+/HPV+ (double positive) disease [33]. The presence of p16 positivity alone has been associated with better survival. For example, Lewis et al. found that p16+/HPV− patients had significantly better OS than p16-negative patients. In the same study, however, OS and DFS rates were comparable in the both groups of HPV-positive patients (p16+/HPV+ and p16+/HPV−) [34]. By contrast, Perrone et al. found that p16-positivity does not necessarily provide a survival advantage; those authors found no difference in OS between p16+ and p16− cases among HPV-negative patients [35]. Sharma et al. showed that 5-year OS was more than 30% lower in the discordant groups (p16+/HPV− and p16−/HPV+) than in the “true” positive group (p16+/HPV+) [24]. Based on these data, it appears that p16 testing alone, without additional complementary assays, may not be specific enough to definitively detect the presence of HPV-associated carcinogenesis. Importantly, immunohistochemistry analysis requires an experienced pathologist to properly interpret the results. Consequently, a single method may be insufficient to accurately determine HPV positivity and therefore a combination of at least two HPV testing methods may be necessary. However, more research is needed, and depending on the results of those studies, it may be necessary to update future editions of the AJCC/UICC TNM staging manual.

Although the 8th edition of the AJCC staging system undoubtedly represents a major improvement over the 7th edition, discrimination between the various stages (in terms of OS) remains imperfect. Several authors have argued that the new TNM classification of OPSCC still demonstrates a less than satisfactory discriminative power. Wuerdemann et al. evaluated 378 cases, finding that the staging system of the 8th edition did not discriminate well between stage II and III HPV-positive patients [36]. By contrast, Gupta et al. found that the clinical and pathological staging in the 8th edition both discriminated between stages better than the 7th edition, although they found no significant differences in 5-year OS between clinical stages I and II when applying the 8th edition criteria. Those authors conclude that stratification was more accurate when based on pathological versus clinical classification [37]. Wuerdemann et al. performed another study to compare adjacent staging groups, finding that the 8th edition criteria discriminated poorly between stages II and III [22]. Van Gysen et al. reported similar findings with regard to the poor discrimination (8th edition) in OS between stages II and III [11].

Several authors have pointed out inconsistencies between clinical and pathological staging in the 8th edition. In the 7th edition, clinical and pathological staging were based on the same criteria, but in the 8th edition, the clinical and pathological lymph node (N) differ: the clinical N stage is based on lymph node size and laterality on radiological findings whereas it is based on the number of nodal metastases on the pathological examination. The reasons for this difference is related to the finding of the ICON-S study, which was the basis for the new staging criteria of HPV-positive OPSCC. The ICON-S study included a patient cohort consisting mainly of patients who underwent primary radiotherapy (98% of cases), versus only 2% who underwent primary surgery [16]. Data from another study of 702 HPV-positive OPSCC patients diagnosed by CT and/or MRI was incorporated into the findings of the ICON-S study to develop a clinical staging system [24]. Interestingly, in that study, the survival rate for patients with pathological stage III disease was based on only 23 patients [38]. For this reason, the survival estimates may not be reliable.

## Conclusions

The 8th edition of the AJCC TNM staging system introduced significant changes to the classification of oropharyngeal cancer. While it remains imperfect in certain areas, the new edition represents a major improvement in group discrimination and risk stratification in patients with HPV-positive OPSCC. In the future, it seems likely that the methods used to assess HPV-positivity will need to be better defined, and the pathological and clinical staging criteria may also need to be unified. Nevertheless, the new staging system is substantially more accurate than the previous system and should be widely adopted in routine clinical practice.
